# History matters

**DOI:** 10.5195/jmla.2017.112

**Published:** 2017-01

**Authors:** Stephen J. Greenberg

Many years ago, two British humorists wrote (facetiously, but not altogether inaccurately) that “History is not what you thought. It is what you can remember. All other history defeats itself” [1]. It is a dilemma that all of us—librarians, archivists, informationists, whatever—have all faced: what do we need to remember, and for how long. When does today become “History,” with a capital H?

History does not record that W. C. Sellar and R. J. Yeatman, authors of *1066 and All That,* ever worked as librarians. However, both were Oxford graduates, and Sellar was a schoolmaster for some years. While their exposition of history was delightfully idiosyncratic, their understanding of memory was spot on.

As information professionals, whatever our exact job title, memory is at the heart of what we do. Our collections, be they analog or digital, stored on shelves or in the Cloud, are the amalgamated memory of our field. Everything will be history someday, and if anything, the pace has quickened. There is something almost plaintive about a product that calls itself “up-to-date,” and what is as out-of-date as yesterday’s Facebook post?

Our responsibilities lie far deeper than that. There are few fields whose development is so deeply tied to its past as the health sciences. History matters to us: we measure our progress from where we started as well as to where we hope to go. It is not a matter of finding some long-forgotten cure in a stereotypical dusty old tome or harming a patient because a journal article that should have been read was neglected because it was “too old” to pop up on a literature search. Research protocols of the present and future are haunted by long-forgotten ghosts of policy and procedure, as any reader of Siddhartha Muhkerjee will know [2]. We are prisoners of the past only when we do not remember that past.

As custodians of that past, we bear the responsibility of collecting, preserving, organizing, making accessible, and (at some level) interpreting the historical materials in our care. To that end and to foster a conversation that will assist us in those tasks, I am proposing a new regular column for the *Journal of the Medical Library Association (JMLA)* to be called “History Matters.” Embedded (I hope!) in that title are two thoughts: a discussion of matters historical as they affect us in our daily work and a reminder that history really does matter to us, the professional memory caretakers in the health sciences.

In 2013, following the work of a task force (suggested by Lucretia W. McClure, AHIP, FMLA) of historical-minded MLA members, Michael Flannery launched “Clio’s Column: Essays on the History of the Health Sciences Libraries, Librarians, and Print Culture,” a feature in the *JMLA,* that was to provide “a regular means of highlighting aspects of our professional and institutional past.” Flannery was quite clear about what he *did not* want for the column: reports of “new research tools or new collections...bibliographies, book reviews, commentaries, editorials, and ‘remember when’ pieces.” Flannery wrote, “the column seeks to cast a wide net and publish a wide range of topics including articles related to collections, people, processes, achievements, challenges, and other items of general [historical] interest to medical librarians” [3].

So the call went out, but the response was meager. In a recent email exchange, Flannery told me he still believes that “the project has tremendous potential,” but sadly, “the response I [that is, Flannery] got was insufficient to sustain a regular series.”

There were some good responses, such as Elisabeth Brander’s April 2015 article on the Henry J. McKellops collection of 760 titles in the history of dentistry, housed in the Bernard Becker Medical Library at Washington University, St. Louis [4]. McKellops was in some ways a shadowy figure, but the collection he compiled was one of the most comprehensive in this country at the time. However, Flannery received few submissions of this caliber.

What I am suggesting, then, is something of a reboot, but with a difference: What interests me, and what I hope will stimulate readers and potential contributors, is not so much the “what” and “where” questions as the matter of “*why?*” It is great that Brander and the Becker Library have an extensive and well-curated collection of early dental classics (which can be pretty scary, especially when illustrated—such books are not for the squeamish), but in this increasingly digital age, why do we, why *should* we, remember them?

And so my new proposal will concentrate on the “why” of what we do: what makes these things “memorable” in the fashion of Sellar and Yeatman; deeper, I hope, and with a different layer of subtlety. It is vital to know what our collections hold, and how to share that information, but this column proposes that *why* we hold these materials is just as important. We want our holdings to be more than just something half-remembered in a locker room.

Individuals who are interested in writing for “History Matters” should contact Stephen Greenberg, MSLS, PhD, greenbes@mail.nih.gov, Coordinator of Public Services, History of Medicine Division, National Library of Medicine, 8600 Rockville Pike, Bethesda, MD 20894.

This work was supported by the Intramural Research Program of the National Institutes of Health, National Library of Medicine.
